# Scrub typhus in Jiangsu Province, China: epidemiologic features and spatial risk analysis

**DOI:** 10.1186/s12879-018-3271-x

**Published:** 2018-08-06

**Authors:** Huiyan Yu, Changkui Sun, Wendong Liu, Zhifeng Li, Zhongming Tan, Xiaochen Wang, Jianli Hu, Shanqiu Shi, Changjun Bao

**Affiliations:** 1 Department of Acute Infectious Disease Control and Prevention, Jiangsu Provincial Center for Disease Control and Prevention, Nanjing, 210009 China; 2 Department of Remote Sensing Imagery, Provincial Geomatics Center of Jiangsu, Nanjing, 210013 China

**Keywords:** Scrub typhus, Spatial epidemiology, ENM, Maxent, GIS

## Abstract

**Background:**

With the increasing incidence of scrub typhus in recent years, it is of great value to analyse the spatial and temporal distribution of scrub typhus by applying micro-geographical studies at a reasonably fine scale, and to guide the control and management.

**Methods:**

We explored the use of maximum entropy modelling method to confirm the spatial and temporal distribution of scrub typhus according to the occurrence locations of human cases in Jiangsu Province. The risk prediction map under specific environmental factors was therefore drawn by projecting the training model across China. The area under the curve and the omission rate were used to validate the model. Meanwhile, Jackknife tests were applied to enumerate the contribution of different environmental variables, then to predict the final model. The predicted results were validated by using China’s known occurrence locations.

**Results:**

A total of 566 occurrence locations with known 4865 scrub typhus occurrence records were used in our study. The number of female cases was higher than male cases, with a proportion of 1.17:1, and people in any age group could be infected. The number of cases presented an inverted-U relation with age. The percentage of cases aged from 60 to 69 years old was the highest, accounting for 30.50% of all cases. Ecological niche modelling results indicated that the locations of scrub typhus cases, which was of great importance in the disease transmission cycle, had a certain ecological niche with environmental elements in many dimensions. Moreover, the key environmental factors for determining scrub typhus occurrence were temperature (including temperature seasonality, min temperature of coldest month, mean diurnal range, and monthly mean temperature), precipitation of wettest month, and land cover types. The risk prediction maps indicated that mid-eastern China was the potential risk areas for scrub typhus of “autumn type”. Meanwhile, in our results, Guangdong Province was the high-risk region for “autumn type” scrub typhus, where cases were mainly reported as “summer type”.

**Conclusion:**

The combination of climatic and geographic factors with GIS methods is an appropriate option to analyse and estimate the spatial and temporal distribution of scrub typhus.

## Background

Scrub typhus is an acute febrile disease, which can be transmitted to humans through infected chigger mites [1]. Before 1986, this kind of disease was only found in southern China, named as “summer type” with an onset period from March to November [2, 3]. In the autumn-winter period of 1986, scrub typhus was found in Shandong Province and Jiangsu Province, China. Since then, the number of people infected with this type of scrub typhus has increased in northern China [3–5]. As for this type, it was mainly found from September to December and peaked in October, being categorized into “autumn type”.

Until recently, the habitat flexibility of chigger mites has not been systematically studied, and thus, the neglect of scrub typhus was unavoidable in most epidemic regions. With the increasing incidence of scrub typhus in recent years, the accurate identification of its occurrence locations has been a crucial problem to be resolved urgently, concerning the clinical diagnosis and control measures [6]. At present, ecological niche modelling (ENM) approach is extensively applied to predict the potential risk areas of species [7–9]. ENMs apply statistical and machine learning theories to analyse the occurrence of one species and to build character functions that estimate the possible shape of its realized niche within the niche space [10]. According to this theory it may be easy to analyze the relevant factors probably influencing species^’^ habitat across the target landscapes [11]. This method has been utilized to characterize the potential risk distribution of species, to understand the effects of climate change, and to predict the high-risk areas of disease occurrence in the fields of ecology, biogeography and evolutionary biology [12–14].

This paper aims to analyse the epidemiologic features and model the spatial and temporal distribution of scrub typhus in China on the basis of climatic and geographic factors by using ENM.

## Methods

### Scrub typhus occurrence data

Jiangsu Province, located in eastern China, was chosen for the study. It has been considered as a main endemic area of scrub typhus. Scrub typhus became a reportable disease in 2006, regulated by China’s National Disease Reporting Information System. Under this guidance, all medical institutions were required to submit the report of scrub typhus cases everyday with unified format including the information about gender, age, residential places, professions and date of disease onset through the web-based surveillance system.

According to the guidebook for prevention and control of scrub typhus (Chinese Center for Disease Control and Prevention, 2008), coincidence of three or more of the following items constituted a clinical case of scrub typhus: (1) a field exposure history 1–3 weeks before illness onset; (2) symptoms including high fever, lymphadenopathy, skin rash, splenomegaly, hepatomegaly, or multiorgan dysfunction; (3) typical cutaneous lesions (eschars or ulcers); (4) rapid defervescence with appropriate antibiotics; and (5) Weil-Felix OX-K agglutination titer ≥1:160. Confirmed cases were defined as clinical cases with a positive result for PCR test targeting a 56-kDa gene of *Orientia tsutsugamushi*, *Orientia tsutsugamushi* isolation or four-fold or higher levels of IgG antibody titers between convalescent-phase and acute-phase sera.

**Fig. 1:**
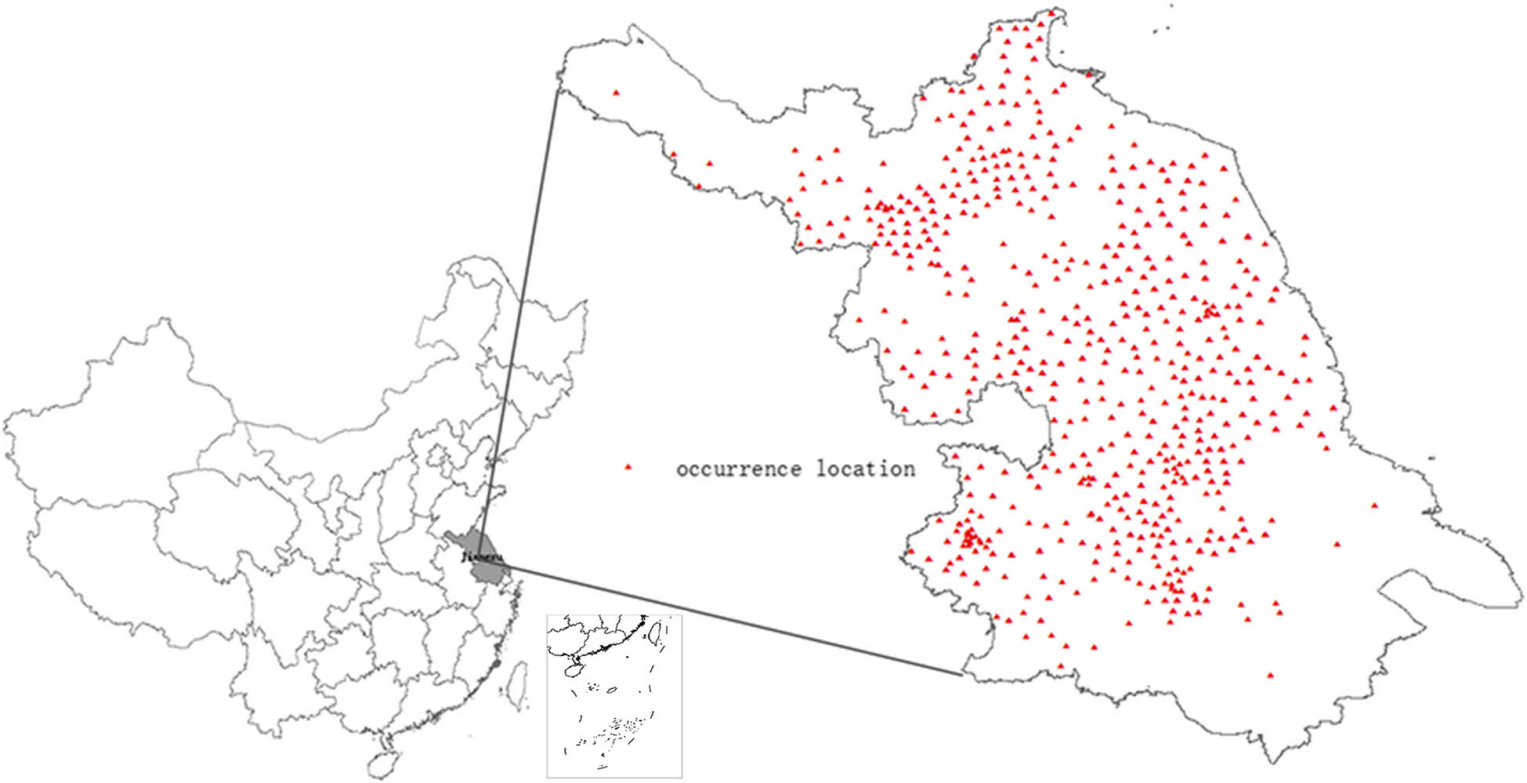
Spatial distribution of scrub typhus cases in Jiangsu Province, 2010–2015

From January 2010 to December 2015 in Jiangsu Province, 566 occurrence locations with 4865 clinical and confirmed cases of scrub typhus were identified. All records were geo-referenced by combining patient’s residential towns and Jiangsu Province vector maps (Fig. 1).

### Environmental factors

Environmental datasets used in this study were downloaded and detailed below.

Seven climatic and 19 bioclimatic variables were collected from the WorldClim database (http://www.worldclim.org, version 2.0) with a resolution of 30 s and had been averaged over a 50-year time period from 1950 to 2000. Derived from the monthly temperature and rainfall, these variables can be utilized to produce more biologically meaningful variables and can be used to reflect more information, such as annual trends, seasonality and extreme or limiting environmental factors for niche modelling.

Elevation data was downloaded from the SRTM mission (http://srtm.csi.cgiar.org/SELECTION/inputCoord.asp) with a resolution of 90 m. The data was used to derive the slope, aspect and composite topographic layers.

The monthly maximum normalized difference vegetation index values (NDVI), enhanced vegetation index (EVI), and land cover types were obtained from Moderate Resolution Imaging Spectroradiometer (MODIS) satellite imagery (https://ladsweb.nascom.nasa.gov/search/). In land cover type, the classes that we selected were defined according to the International Geosphere-Biosphere Program (IGBP) 17-class scheme. This set of cover types includes 11 categories of natural vegetation covers broken down by life form, 3 classes of developed and mosaic lands, and 3 classes of non-vegetated lands.

All the environmental layers were resampled into the same projection information and were converted into the ASCII raster data format required by ENM.

### Ecological niche modelling

Ecological niche modelling, using maximum entropy algorithm (Maxent, version 3.3.3 k), was adopted to predict the distribution of scrub typhus. It is a multi-purpose machine learning program that can achieve high predictive accuracy and enjoys several additional attractive properties [15, 16]. With a simple and precise mathematical formulation, it can be used to estimate the probability distribution of a species based on presence-only datasets and environmental constraints [17–19].

Maxent was applied accordingly with the following changes in model run. 75% were selected at random to construct the model, while the remaining were used for validation. Regularization multiplier was set to 1. The background points were selected randomly by removing the occurred position within the study area, and the maximum number of background points was kept at 10000. In order to obtain a robust model, we ran 10 replicates based on independent random partitions, then averaged the results. Replicated run type was set to cross-validate. Maximum iterations were set to 5000, with convergence threshold of 1 × 10^− 6^. Auto features of environmental variables were selected. Response curves were used to analyse the suitable range of each environmental condition for the occurrence of scrub typhus. The logistic output format of the predictive map with probability values ranging from 0 (unsuitable) to 1 (suitable) was chosen to visualize the potential risk of scrub typhus. The default prevalence parameter was set to 0.5 as the risk cut-off to distinguish potential presence regions from potential absence regions.

The threshold-dependent binomial test, using the extrinsic omission rate as statistic, was carried out to detect the statistical significance of the model [20, 21]. Jackknife procedure and the percentage of variables’ contributions were applied to evaluate the relative importance of each environmental variables. The area under the receiver operating characteristic curve (ROC), known as the area under the curve (AUC), was used to evaluate the prediction performance of the model [22–24]. Generally, AUC values of 0.5–0.7 were considered as low accuracy, values of 0.7–0.9 were considered as useful applications and values of > 0.9 were considered as high accuracy [25].

### Model projection

A potential risk map of China was produced by projecting the training model gained from Jiangsu Province onto the specific environmental layers. Meanwhile, the model performs was evaluated using the known occurrence locations of disease.

## Results

### Epidemiologic features of confirmed cases

**Fig. 2:**
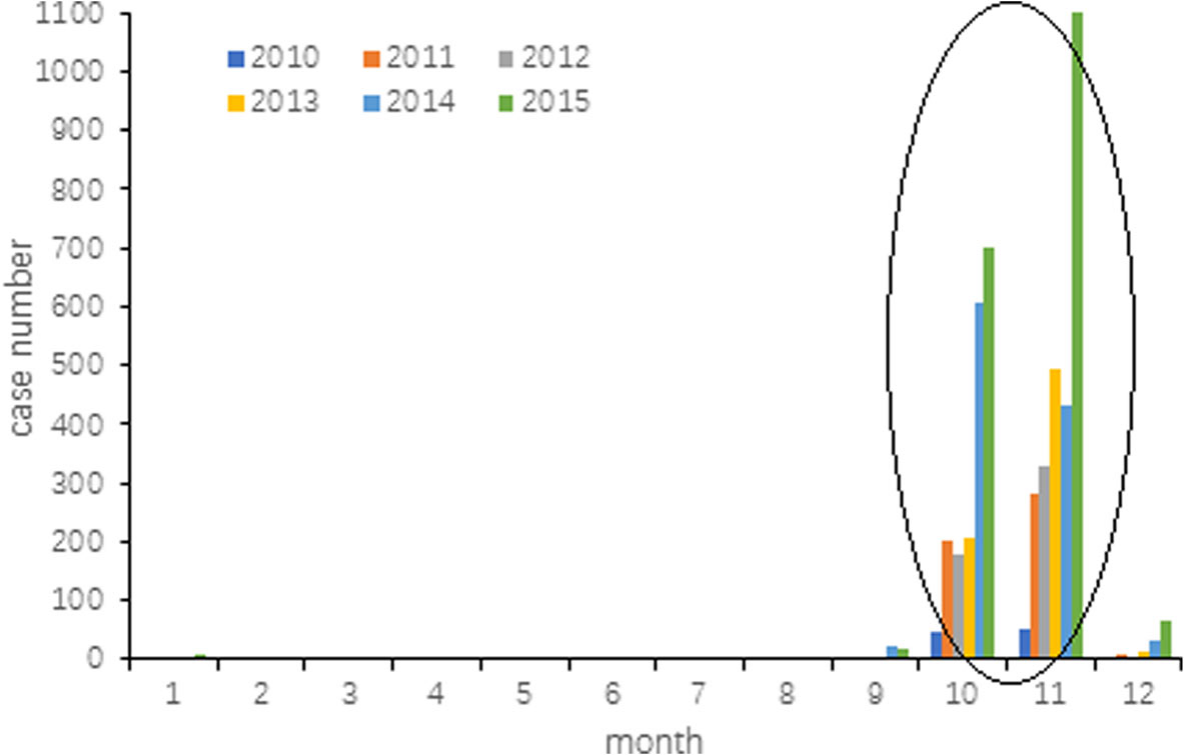
Seasonal distribution of confirmed cases in Jiangsu Province, 2010–2015

**Fig. 3:**
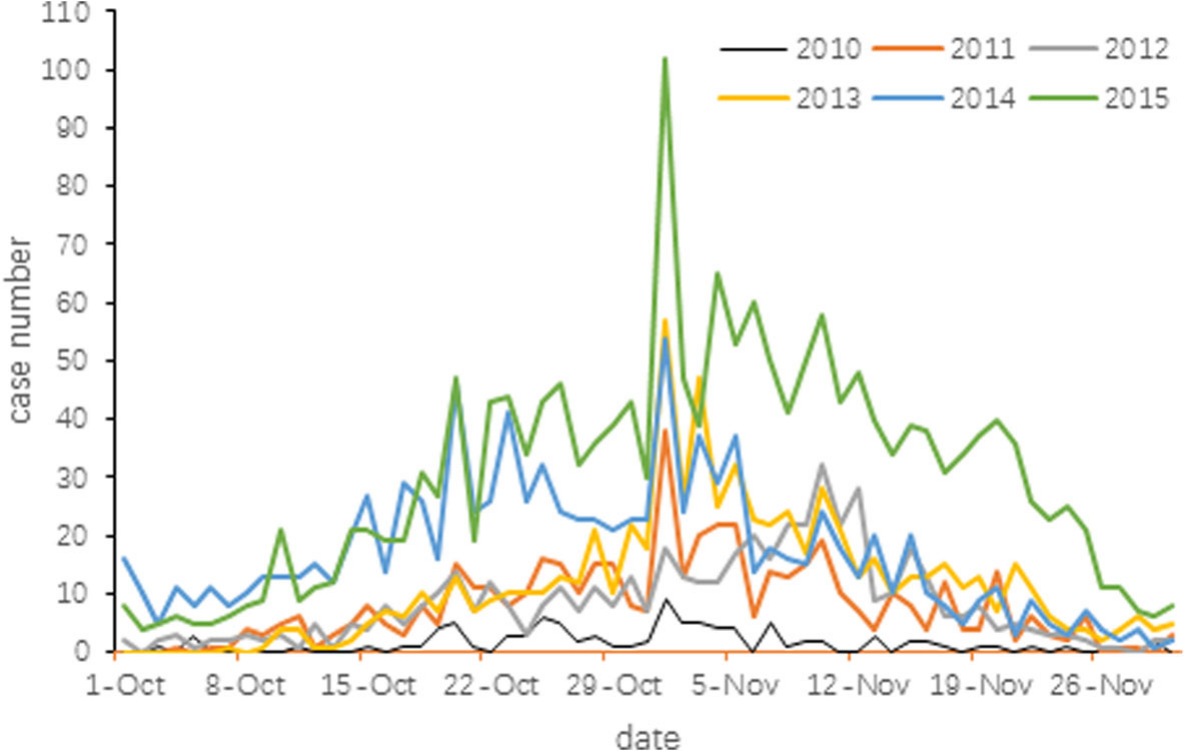
Temporal distribution of cases in October and November in Jiangsu Province, 2010–2015

There were 97、497、525、713、1109、1924 clinical and confirmed cases of scrub typhus in Jiangsu Province from 2010 to 2015. Cases were clustered in autumn with a single incidence peak appearing from October or November (Fig. 2). Temporal distribution characteristics of scrub typhus occurring in October and November from 2010 to 2015, were listed in Fig. 3. Time trend analysis revealed that cases started to appear from early October. Case numbers then slowly rose and peaked in early November, followed by a decline and finally reached the lowest numbers at the end of November. Peak incidence usually occurs between October 20th and November 10th.

Table 1 listed the demographic characteristics of scrub typhus cases in Jiangsu Province, from 2010 to 2015. According to this study, the number of cases increased year by year. On the whole, the number of female cases was higher than male cases, with a proportion of 1.17:1, and people in any age group could be infected by *Orientia tsutsugamushi*. The number of cases presented an inverted-U relation with age. Those who aged from 60 to 69 years old accounted for 30.50% of all cases, with the highest percentage. For different occupations, epidemiologic surveillance indicated that the susceptible population of scrub typhus were farmers.

**Table 1:** Demographic characteristics of scrub typhus cases in Jiangsu Province, 2010–2015

Features	2010 *n*(%)	2011 *n*(%)	2012 *n*(%)	2013 *n*(%)	2014 *n*(%)	2015 *n*(%)	Total *n*(%)
Sex							
Male	39(40.21)	233(46.88)	268(51.05)	292(40.95)	498(44.91)	914(47.51)	2224(46.13)
Female	58(59.79)	264(53.12)	257(48.95)	421(59.05)	611(55.09)	1010(52.49)	2621(53.87)
Age							
0~ 9	4(4.12)	14(2.82)	14(2.67)	7(0.98)	14(1.26)	13(0.68)	66(1.36)
10~ 19	0	3(0.60)	1(0.19)	3(0.42)	10(0.90)	10(0.52)	27(0.55)
20~ 29	1(1.03)	12(2.41)	12(2.29)	15(2.10)	34(3.07)	42(2.18)	116(2.38)
30~ 39	5(5.15)	23(4.63)	26(4.95)	34(4.77)	56(5.05)	77(4.00)	221(4.54)
40~ 49	13(13.40)	71(14.29)	96(18.29)	110(15.43)	166(14.97)	288(14.97)	744(15.29)
50~ 59	32(32.99)	130(26.16)	122(23.24)	177(24.82)	254(22.90)	457(23.75)	1172(24.09)
60~ 69	33(34.02)	147(29.58)	149(28.38)	227(31.84)	333(30.03)	595(30.93)	1484(30.50)
70~ 79	7(7.22)	77(15.49)	85(16.19)	103(14.45)	198(17.85)	355(18.45)	825(16.96)
80~ 100	2(2.06)	20(4.02)	20(3.81)	37(5.19)	44(3.97)	87(4.52)	210(4.32)
Occupation							
Worker	3(3.09)	19(3.82)	19(3.62)	31(4.35)	42(3.79)	60(3.12)	174(3.58)
Retiree	3(3.09)	16(3.22)	18(3.43)	27(3.79)	40(3.61)	85(4.42)	189(3.88)
Student& children	4(4.12)	18(3.62)	17(3.24)	10(1.40)	22(1.98)	20(1.04)	91(1.87)
Farmer	82(84.54)	413(83.10)	441(84.00)	619(86.82)	946(85.30)	1635(84.98)	4136(85.02)
Unemployed	2(2.06)	16(3.22)	12(2.29)	14(1.96)	43(3.88)	64(3.33)	151(3.10)
Teacher	2(2.06)	3(0.60)	6(1.14)	4(0.56)	8(0.72)	9(0.47)	32(0.66)
Others	1(1.03)	12(2.41)	12(2.29)	8(1.12)	8(0.72)	51(2.65)	92(1.89)

%: constituent ratio

### Variation of environmental factors

Some highly intercorrelated (correlation coefficient > 0.9 or < − 0.9) variables were removed as they might violate statistical assumptions and alter model predictions [26]. Finally, 17 environmental variables, including 3 climatic factors, 9 bioclimatic factors, 2 surface factors, and 3 topographic factors, were used in constructing species distribution model. Table 2 listed the appropriate range of each environmental factor for scrub typhus occurrence and the logistic output probability of scrub typhus presence with a cut-off point of 0.5. It indicated that scrub typhus occurrence had a specific ecological niche with multi-dimensional environmental factors, which play important roles in its transmission cycle.

**Table 2:** The suitable range and percent contribution of each environmental condition for scrub typhus occurrence

Variable	Description(unit)	Suitable range^a^	Percent contribution^d^
BIO_01	Annual mean temperature(°C)	11.3–14.3	1.8
BIO_02	Mean diurnal range	8.7–10.7	14.5
BIO_04	Temperature seasonality	920.0–980.0	24.9
BIO_05	Max temperature of warmest month(°C)	31.7–32.8	1.1
BIO_06	Min temperature of coldest month(°C)	−4.2 to-1.2	19.9
BIO_12	Annual precipitation (mm)	740.0–1000.0	3.6
BIO_13	Precipitation of wettest month (mm)	196.0–275.0	8.7
BIO_14	Precipitation of driest month (mm)	17.0–28.0	2.0
BIO_15	Precipitation seasonality (mm)	58.0–82.0	3.6
prec	Precipitation (mm)	22.0–71.0	0.5
tavg	Monthly average temperature (°C)	8.9–11.1	5.6
wind	Wind speed (m s-1)	2.2–2.5	1.6
NDVI	Normalized difference vegetation index	0.18–0.39	7.5
LC	Land cover type	7,13,14^b^	4.7
DEM	Altitude(m)	none^c^	0.0
Aspect	Aspect	none^c^	0.0
Slope	Slope	none^c^	0.0

^a^The suitable range of each variable indicates the conditions within which the probability of scrub typhus occurrence is higher than 50%

^b^7: Open shrublands; 13: Urban and Built-up; 14: Cropland-Natural Vegetation Mosaic. The combination of these classes could be found in and around the countryside

^c^Nonemeans this environmental factor has little effect on the final model construction

^d^The percentage contribution illustrates the relative contributions of the environmental variables to the final training Maxent model using the averages of the repeated 10 runs

**Fig. 4:**
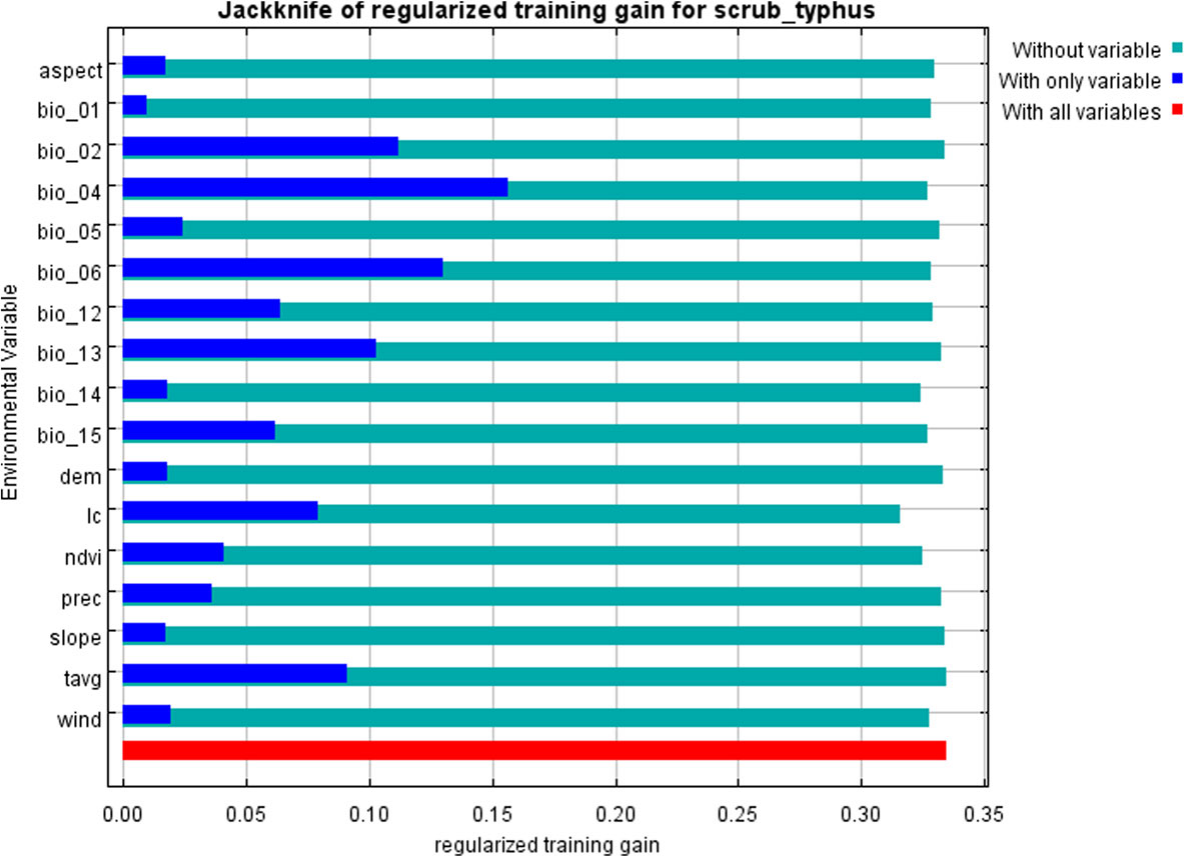
Results of the Jackknife test of variable importance

The relative importance of environmental elements on scrub typhus occurrence based on Jackknife test was shown in Fig. 4 and Table 2. This indicated that the critical environmental factors for determining scrub typhus occurrence were temperature (including temperature seasonality, min temperature of coldest month, mean diurnal range, and monthly mean temperature), precipitation of wettest month, and land cover types.

### Statistical analysis and model evaluation

**Fig. 5:**
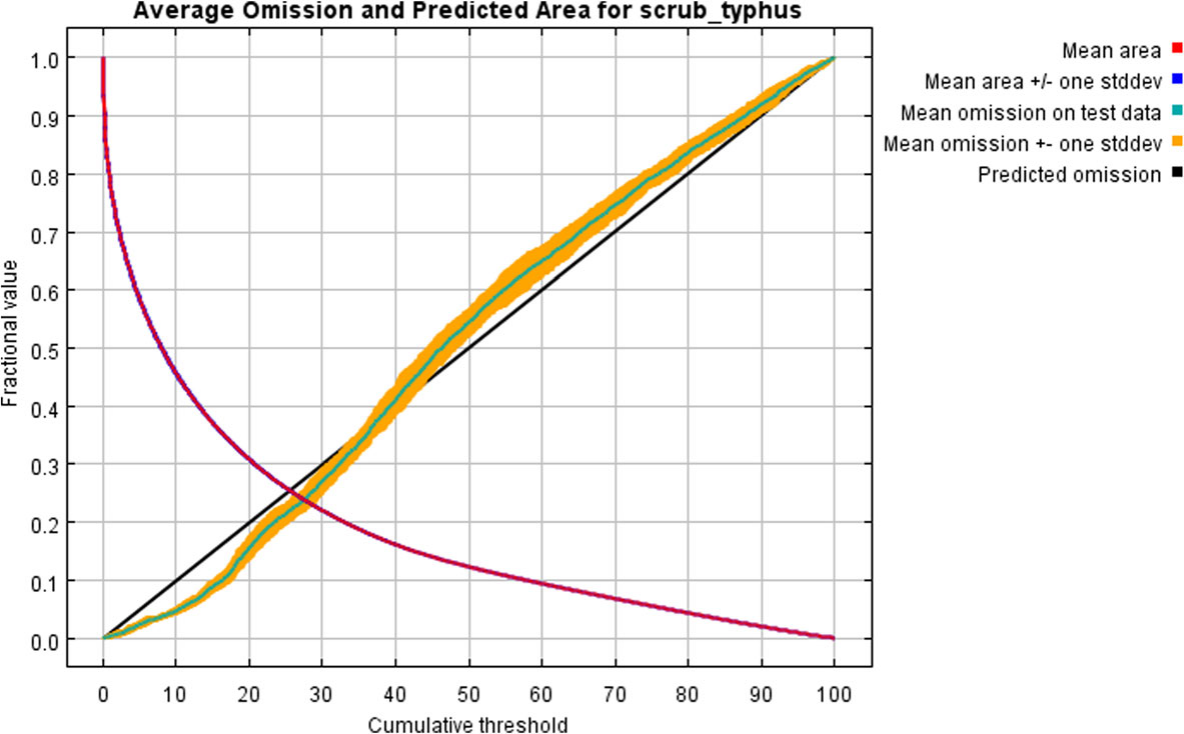
Omission and predicted areas for scrub typhus

The average omission and predicted area for scrub typhus varied with different cumulative threshold (Fig. 5). The results showed that the omission on test and training samples was very consistent with the predicted omission rate. Thus, the training model had a good statistical significance.

The omission rate with the *p*-value calculated by binomial omission tests, and the AUC of the testing and training model was shown in Table 3. All 10 single runs were statistically significant, and AUC for all 10 models were higher than 0.80, indicating that both the testing model and the training model performed well in predicting the potential high-risk areas of scrub typhus.

**Table 3:** Model evaluation results of each single run

Run	Training AUC	Testing AUC	Training omission rate^a^	Testing omission rate	*P* value
1	0.833	0.827	0.009	0.017	< 0.0001
2	0.832	0.829	0.008	0.005	< 0.0001
3	0.832	0.833	0.011	0.022	< 0.0001
4	0.833	0.827	0.009	0.010	< 0.0001
5	0.833	0.828	0.009	0.012	< 0.0001
6	0.832	0.841	0.008	0.010	< 0.0001
7	0.833	0.835	0.008	0.005	< 0.0001
8	0.833	0.822	0.009	0.022	< 0.0001
9	0.832	0.833	0.011	0.010	< 0.0001
10	0.834	0.818	0.008	0.015	< 0.0001

^a^Balance training omission, predicted area and the threshold value

### Potential risk areas of scrub typhus

**Fig. 6:**
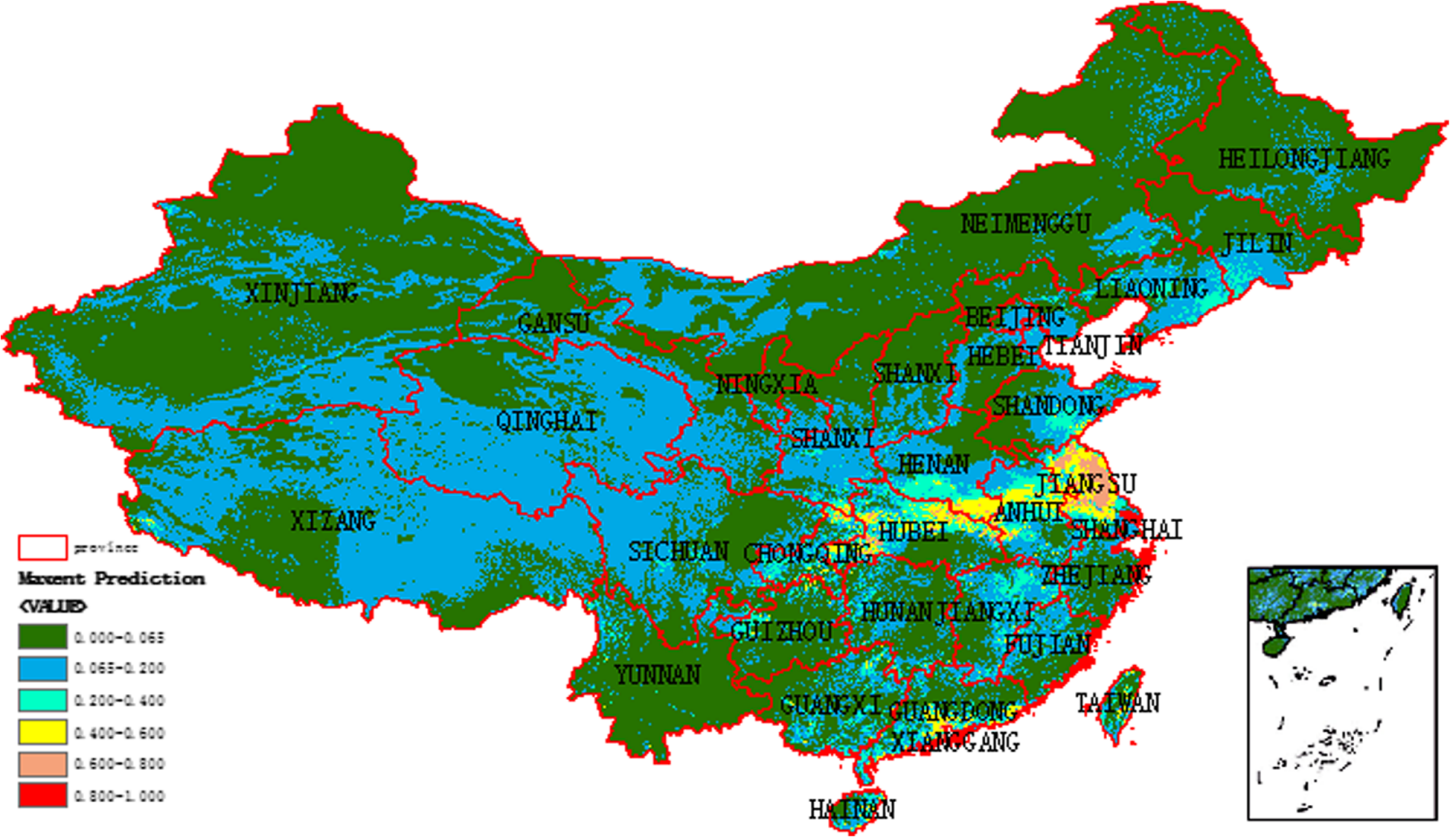
Predicted potential risk areas for scrub typhus in November in China

**Fig. 7:**
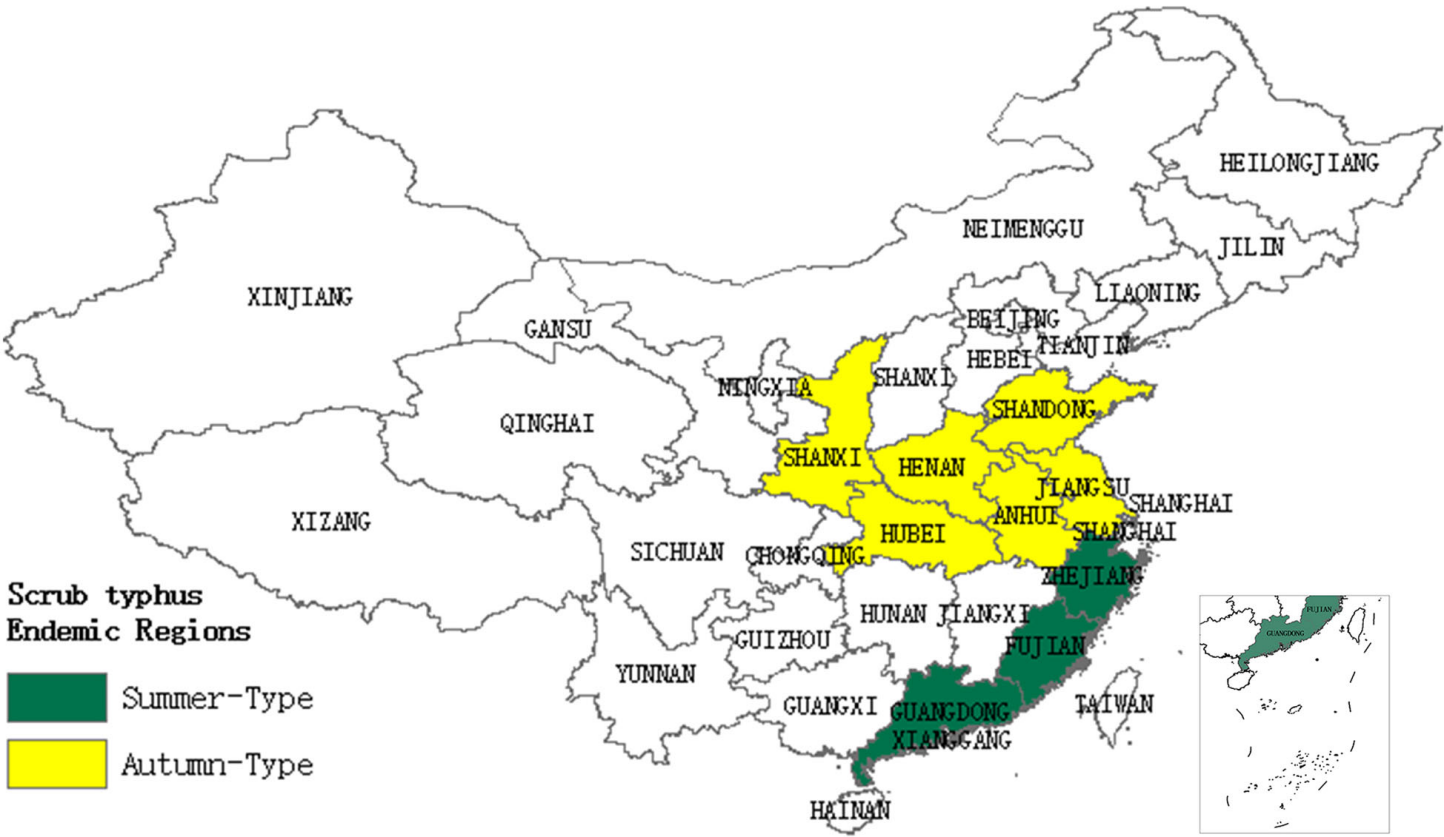
Spatial distribution of reported cases of scrub typhus in China

We made a prediction about potential high-risk regions of scrub typhus in November in China, since the case numbers of scrub typhus in Jiangsu Province peaked in November each year. The predicted potential high-risk regions, with a cut-off point of 0.065 for the logistic output probability, were shown in Fig. 6. The known occurrence [27, 28] of the disease with “summer type” and “autumn type” in China was shown in Fig. 7. The potential high-risk regions of “autumn type” for scrub typhus were mainly distributed in Shandong Province, Jiangsu Province, Anhui Province, Henan Province, and Hubei Province. Within the predicted potential risk regions, most of them were recognized by the known cases of scrub typhus. However, still some predicted high-risk areas remained to be verified in the future.

## Discussion

As an infectious disease with natural foci, it is feasible to predict the potential high-risk areas of scrub typhus by analysing environmental elements [29]. This study aimed at modelling and predicting the potential risk distribution of scrub typhus. In our research, we first applied ENM to analyse the data of both spatial scrub typhus cases and its habitat, and to predict the potential risk areas. Then latent at-risk areas of whole China were estimated.

Recent studies indicate that scrub typhus occurs much more frequently in secondary vegetation, where chiggers and small mammal hosts are more abundant [33]. Epidemiologic surveillance shows that the infection rate among farmers are exceptionally high, mainly due to their frequent contact with disturbed habitats. Another fact is that scrub typhus infection is more common in female than male with similar variation tendency. It may be because most women generally spend more time on farm work outdoors. Global trends in population aging can be witnessed currently. Consequently, more and more young people go to cities to earn the family’s bread and leave the elderly in rural areas to take care of their children. These phenomena may contribute to a higher percentage of the elderly patients. The incidence of scrub typhus has obvious seasonal character. During the research period, scrub typhus cases occurred exclusively every year from September to December. From the model, we found that temperature was the key environmental factor with a suitable range for scrub typhus occurrence. This was consistent with the recent studies [30–33]. In general, temperature plays an important role in the spread of vector-borne diseases [34]. It can affect the replication and dissemination of the pathogen directly within an appropriate scale [35], the ecological dynamics of virus vectors [36], and the behaviour and ecological characteristics of both wild and native hosts [35].

The potential high-risk map in China suggests that the broad central coast of China is high-risk areas for “autumn type”. Besides, in our results, Guangdong Province, where cases mainly reported as “summer type”, is also a high-risk region for “autumn type”. Therefore, epidemiological surveillance should be performed in these areas in the future.

ENM method is an especially effective method to predict the potential risks of a disease [37–39], as identifying the potential risk distribution of human disease is a very complex task [40]. Among the numerous ENM methods, Maxent has been identified as the best algorithm [41, 42]. It is clarified that the projection of the model into geographic space represents the potential areas at the risk of the disease, not the actual or realized occurrence locations. So, predictions made from models should not provide a complete substitute for detailed field data, thus, to test the prediction result of our model, surveillance in those places predicted to be at high risk for scrub typhus occurrence but without recorded human cases to date should be enhanced.

## Conclusions

This research, for the first time, provided a method for predicting the potential risk distribution of scrub typhus by using ecological requirements. The results showed that the incidence of scrub typhus had obvious seasonal character, and mid-eastern China with temperature at a suitable range was the potential risk areas for scrub typhus occurrence with “autumn type”. Meanwhile, other regions, such as Guangzhou Province which was reported as “summer type”, can also be defined with “autumn type”.

Our research showed that the combination of habitat data and field investigation can be used to analyse and predict the potential areas at risk of scrub typhus.

## Abbreviations

AUC: area under the curve
ENM: ecological niche model
GIS: Geographical information system
IGBP: International Geosphere-Biosphere Program
MODIS: Moderate Resolution Imaging Spectroradiometer
NDVI: normalized difference vegetation index
ROC: receiver operating characteristic curve
SRTM: Shuttle Rader Topography Mission
